# Society Meetings

**Published:** 1910-07

**Authors:** 


### SOCIETY MEETINGS.

The American Medical Association appears to have had one of its better meetings at Saint Louis, last month. The attendance was large and the scientific work done was excellent. The social part of the entertainment was all that could be desired. Dr. Walter B. Dorsett, chairman of the committee of arrangements, handled the visitors so well that no complaint was made against the management, which means the highest kind of praise. Buffalo should look well to this matter when the association meets here, as it is likely to in 1912. The chairman of the committee of arrangements is the most important local officer to be chosen wherever the association meets.

---

The twelfth annual conference of the American Hospital Association will be held at the Planters Hotel, St. Louis, September

20, 21, 22 and 23, 1910. An elaborate preliminary program already has been issued and the meeting will prove interesting, as well as instructive.

---

The Medical Society of the County of Erie held its regular meeting, Monday, June 20, 1910, at 8.15 P. M., in the Buffalo Library Building. The program was 1, Stereopticon Night; 2, Reports of Committees; 3, Collation. Dr. Grover W. Wende is the president and Dr. Franklin C. Gram is the secretary.

